# Simple Symptom-Based Prediction of COVID-19: A Single-Center Study of Outpatient Fever Clinic in Japan

**DOI:** 10.7759/cureus.36614

**Published:** 2023-03-24

**Authors:** Shinji Inaba, Yasuhisa Nakao, Shuntaro Ikeda, Yuki Mizumoto, Takeshi Utsunomiya, Masahiko Honjo, Yasutsugu Takada, Naoyuki Nogami, Eiichi Ishii, Osamu Yamaguchi

**Affiliations:** 1 Department of Cardiology, Pulmonology, Hypertension and Nephrology, Ehime University Graduate School of Medicine, Toon, JPN; 2 Department of Community Medicine, Pulmonology and Cardiology, Ehime University Graduate School of Medicine, Toon, JPN; 3 Department of Cardiology, Imabari City Medical Association General Hospital, Imabari, JPN; 4 Department of Surgery, Imabari City Medical Association General Hospital, Imabari, JPN; 5 Department of Hepato-Billiary-Pancreatic Surgery, Ehime University Graduate School of Medicine, Toon, JPN; 6 Department of Pediatrics, Imabari City Medical Association General Hospital, Imabari, JPN

**Keywords:** general public health, clinical diagnosis of covid-19, covid-19 symptoms, outpatient fever clinic, covid-19

## Abstract

Introduction: Coronavirus disease 2019 (COVID-19) symptoms are not fully understood in non-hospitalized individuals in Japan, and COVID-19 differentiation by symptoms alone remained challenging. Therefore, this study aimed to examine COVID-19 prediction from symptoms using real-world data in an outpatient fever clinic.

Methods: We compared the symptoms of COVID-19-positive and negative patients who visited the outpatient fever clinic at Imabari City Medical Association General Hospital and tested for COVID-19 from April 2021 to May 2022. This retrospective single-center study enrolled 2,693 consecutive patients.

Results: COVID-19-positive patients had a higher frequency of close contact with COVID-19-infected patients compared with COVID-19-negative patients. Moreover, patients with COVID-19 had high-grade fever at the clinic compared with patients without COVID-19. Additionally, the most common symptom in patients with COVID-19 was sore throat (67.3%), followed by cough (62.0%), which was approximately twice as common in patients without COVID-19. COVID-19 was more frequently identified in patients having a fever (≥37.5℃) with a sore throat, a cough, or both. The positive COVID-19 rate reached approximately half (45%) when three symptoms were present.

Conclusion: These results suggested that COVID-19 prediction by combinations of simple symptoms and close contact with COVID-19-infected patients might be useful and lead to recommendations for testing of COVID-19 in symptomatic individuals.

## Introduction

Severe acute respiratory syndrome coronavirus 2 (SARS-CoV-2) has continued to rapidly spread while increasing its infectivity since the first coronavirus disease 2019 (COVID-19) case was reported in Japan on January 15, 2020 [1]. Early diagnosis and isolation are essential to control the spread of infection because delayed diagnosis leads to the spread of infection by contacts [2]. Various studies have developed diagnostic scores for COVID-19 by symptoms, but the scores are complex and are, therefore, not used by the general public [3-7]. Additionally, the actual COVID-19 diagnosis status at outpatient fever clinics for non-hospitalized individuals is rarely reported in Japan. Therefore, this study aimed (1) to clarify the symptoms of patients with COVID-19 in a fever outpatient setting and (2) to identify the symptoms for COVID-19 prediction that are simple and accessible to the general public.

## Materials and methods

**Figure 1:**
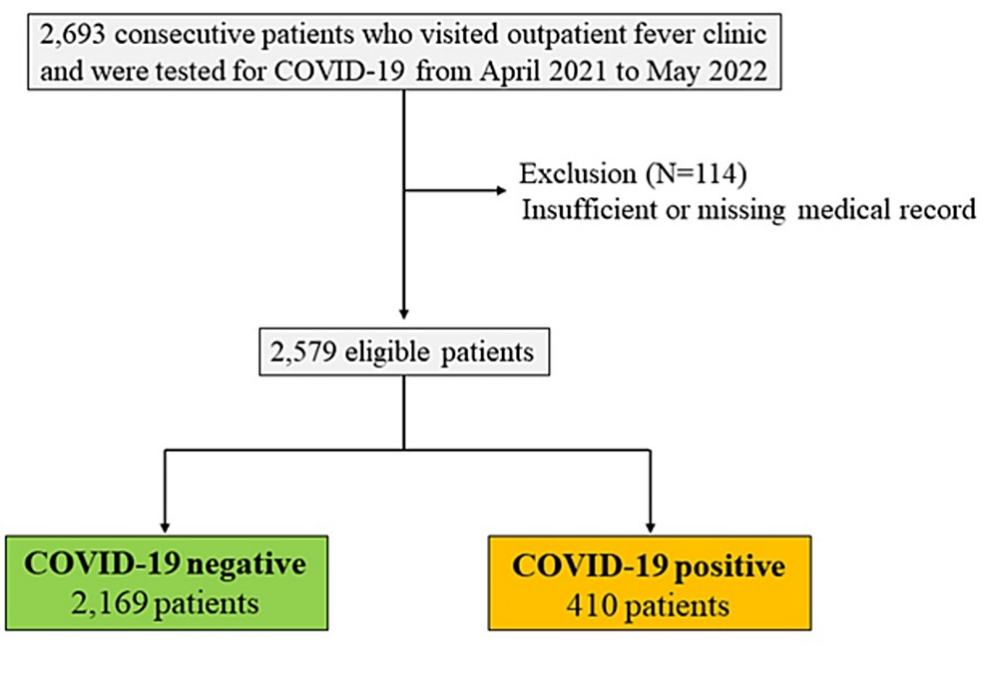
Flow chart of eligible patient

This retrospective single-center study enrolled 2,693 consecutive patients who visited the outpatient fever clinic at Imabari City Medical Association General Hospital and tested for COVID-19 from April 2021 to May 2022 in Japan. This study was approved by the research ethics committee of Ehime University Graduate School of Medicine (#2201006). Among 2,693 patients, we excluded 114 patients with insufficient or missing medical records. Finally, the present study registered 2,579 patients (Figure 1).

COVID-19 diagnosis was made by polymerase chain reaction test or/and antigen kit from nasopharyngeal swabs. High-risk exposure is defined as having had face-to-face contact with a COVID-19 case within 1 meter and >15 minutes. Low-risk exposure is defined as having had contact with a COVID-19 case in a closed environment or within 1 meter for <15 minutes. Vaccination history was determined to be at least one COVID-19 vaccination.

Statistical analysis

Categorical variables were presented as n (%) and continuous variables were expressed as median and interquartile ranges. Categorical variables were compared using the Chi-square test. The Mann-Whitney U test was applied for continuous variables. P values of <0.05 were considered statistically significant. We used logistic regression analysis for detecting COVID-19-positive cases to calculate the odds ratio and their 95% confidence intervals. We selected all covariates for multivariate analyses. Statistical analyses were performed using EZR (Saitama Medical Center, Jichi Medical University, Saitama, Japan) [8].

## Results

**Figure 2:**
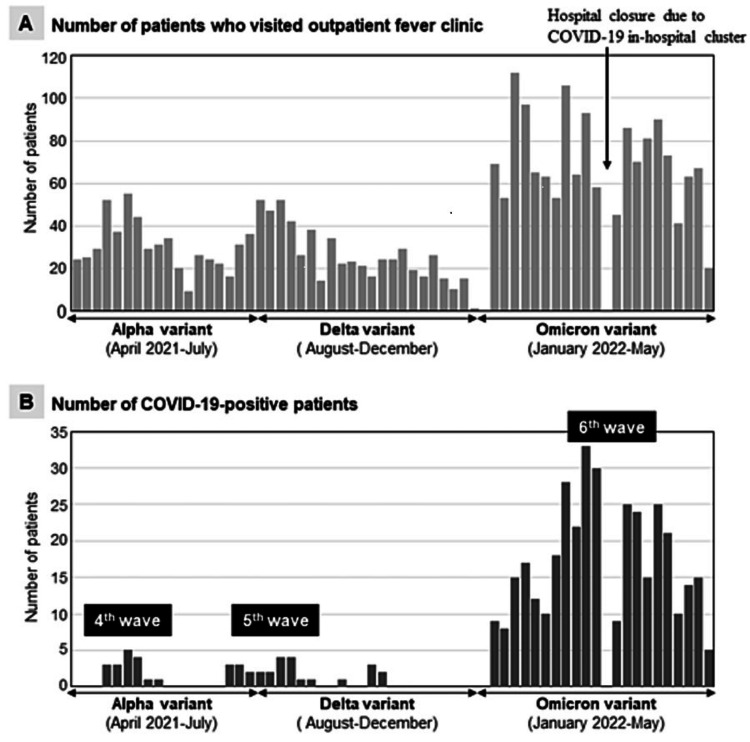
Number of patients who visited outpatient fever clinic (A) and COVID-19-positive patients (B) This hospital is one of four hospitals that diagnose and treat COVID-19 in the Imabari area, the second most populous in Ehime Prefecture, and the results of this analysis should largely reflect the situation in the Ehime Prefecture.

The median age of patients with COVID-19 was 35 years old, and 51.7% were males. The COVID-19-positive rate in the outpatient clinic was 15.9%. The occurrence of COVID-19 in our outpatient fever clinic is shown in Figures 2A, 2B.

The number of fever outpatient visits and COVID-19-positive patients remained mostly parallel. During the study period, 89% (364/410) COVID-19-positive patients occurred during the 6th wave in the Omicron strain epidemic.

Patients with COVID-19 had a higher frequency of high-risk exposure to patients with COVID-19 compared with patients without COVID-19 (47.1% vs. 3.1%, P < 0.001). Conversely, the COVID-19 vaccination history was significantly lower in patients with COVID-19 than that in those without COVID-19 (67.7% vs. 76.3%, P = 0.001) (Table 1).

**Table 1:** Comparison of patient background between positive and negative test results for COVID-19 Values are median (interquartile range) or % (number of observations/total number of patients).

	COVID-19 positive (n=410)	COVID-19 negative (n=2169)	P value
Age, years	35 (19, 48)	35 (20, 54)	0.068
Sex, male	51.7% (212/410)	52.9% (1148/2169)	0.69
High-risk exposure with COVID-19 patient	47.1% (193/410)	3.1% (67/2169)	<0.001
History of COVID-19 vaccination	67.7% (249/368)	76.3% (940/1232)	0.001
Low-risk exposure with COVID-19 patient	5.6% (23/410)	4.3% (93/2169)	0.29
History of out-of-prefecture movement	8.5% (35/410)	8.0% (174/2169)	0.80

Patients with COVID-19 had higher fevers at clinic compared with patients without COVID-19 (37.7℃ [37.0, 38.3] vs. 37.3℃ [36.8, 38.1], P < 0.001). The most common symptom in patients with COVID-19 was sore throat (67.3%), followed by cough (62.0%), which was approximately twice as common as in patients without COVID-19. A loss of taste and smell were present in only 1.7% and 1.0% of COVID-19-positive patients, respectively (Table 2).

**Table 2:** Comparison of symptoms between COVID-19-positive and COVID-19-negative patients Values are median (interquartile range) or % (number of observations/total number of patients).

	COVID-19 positive (n=410)	COVID-19 negative (n=2169)	P value
Temperature at clinic (℃)	37.7 (37.0, 38.3)	37.3 (36.8, 38.1)	<0.001
-Fever (≥37.5℃ at clinic)	59.0% (235/398)	45.9% (992/2161)	<0.001
Sore throat	67.3% (276/410)	38.4% (833/2169)	<0.001
Cough	62.0% (254/410)	30.2% (654/2169)	<0.001
Diarrhea	4.9% (20/410)	15.3% (332/2169)	<0.001
Nausea/vomiting	4.4% (18/410)	15.4% (334/2169)	<0.001
Abdominal pain	3.2% (13/410)	8.9% (192/2169)	<0.001
Loss of appetite	7.3% (30/410)	12.7% (275/2169)	0.003
Sputum	22.4% (92/410)	16.7% (362/2169)	0.006
Cystitis symptom	0% (0/410)	1.8% (38/2169)	0.013
Arthralgia	9.8% (40/410)	6.3% (137/2169)	0.016
Gastralgia	2.0% (8/410)	4.1% (90/2169)	0.046
Shortness of breath	4.1% (17/410)	6.7% (145/2169)	0.067
Asymptomatic	4.1% (17/410)	6.6% (144/2169)	0.072
Constipation	0% (0/410)	0.8% (17/2169)	0.14
Myalgia	3.2% (13/410)	2.2% (48/2169)	0.32
Chills	5.6% (23/410)	6.8% (147/2169)	0.44
Headache	36.8% (151/410)	35.0% (760/2169)	0.52
Fatigue	25.1% (103/410)	25.7% (557/2169)	0.86
Loss of smell	1.0% (4/410)	0.8% (18/2169)	1.0
Loss of taste	1.7% (7/410)	1.7% (37/2169)	1
Runny nose/nasal congestion	33.2% (136/410)	33.1% (719/2169)	1

The logistic regression analyses for detecting COVID-19 positives are shown in Tables 3, 4.

**Table 3:** Correlation of patient background to COVID-19 positive prediction CI, confidence interval.

	Univariate analysis		Multivariate analysis	
	Odds ratio (95% CI)	P value	Odds ratio (95% CI)	P value
High-risk exposure with COVID-19 patient	27.9 (20.4–38.1)	<0.001	23.1 (16.0–33.3)	<0.001
Age, years	0.99 (0.99–1.00)	<0.05	1.01 (1.01–1.02)	<0.001
History of COVID-19 vaccination	0.65 (0.50–0.84)	<0.001	0.63 (0.45–0.89)	<0.01
Low-risk exposure with COVID-19 patient	1.33 (0.83–2.12)	0.24	1.96 (1.16–3.32)	<0.05
History of out-of-prefecture movement	1.07 (0.73–1.56)	0.73	1.48 (0.92–2.39)	0.10
Sex, male	0.95 (0.77–1.18)	0.65	1.21 (0.92–1.60)	0.18

**Table 4:** Correlates of COVID-19 symptom positive prediction CI, confidence interval.

	Univariate analysis		Multivariate analysis （Adjusted for age and gender）	
	Odds ratio (95% CI)	P value	Odds ratio (95% CI)	P value
Fever (≥37.5°C at clinic)	1.70 (1.37–2.11)	<0.001	2.26 (1.76–2.89)	<0.001
Sore throat	3.30 (2.64–4.13)	<0.001	2.81 (2.17–3.63)	<0.001
Cough	3.77 (3.03–4.70)	<0.001	4.41 (3.37–5.79)	<0.001
Runny nose/nasal congestion	1.00 (0.80–1.25)	0.99	0.52 (0.40–0.69)	<0.001
Nausea/vomiting	0.25 (0.16–0.41)	<0.001	0.43 (0.25–0.73)	<0.01
Arthralgia	1.60 (1.11–2.32)	<0.05	1.75 (1.14–2.68)	<0.05
Diarrhea	0.28 (0.18–0.45)	<0.001	0.54 (0.32–0.90)	<0.05
Shortness of breath	0.60 (0.36–1.01)	0.054	0.54 (0.31–0.94)	<0.05
Loss of appetite	0.54 (0.37–0.81)	<0.01	0.65 (0.42–1.01)	0.053
Myalgia	1.45 (0.78–2.70)	0.24	1.60 (0.80–3.17)	0.18
Abdominal pain	0.34 (0.19–0.60)	<0.001	0.65 (0.34–1.22)	0.18
Sputum	1.44 (1.12–1.87)	<0.01	0.86 (0.63–1.16)	0.32
Headache	1.08 (0.87–1.35)	0.49	1.12 (0.87–1.45)	0.39
Fatigue	0.97 (0.76–1.24)	0.81	1.11 (0.84–1.47)	0.47
Asymptomatic	0.61 (0.36–1.02)	0.058	1.23 (0.67–2.26)	0.51
Chills	0.82 (0.52–1.29)	0.38	0.89 (0.54–1.48)	0.65
Loss of smell	1.18 (0.40–3.50)	0.77	1.38 (0.33–5.76)	0.66
Gastralgia	0.46 (0.22–0.95)	<0.05	1.07 (0.48–2.39)	0.87
Loss of taste	1.00 (0.44–2.26)	1.00	1.09 (0.37–3.18)	0.88
Cystitis symptom	0.00 (0.00–)	0.97	0.00 (0.00–)	0.97
Constipation	0.00 (0.00–)	0.97	0.00 (0.00–)	0.98

**Figure 3:**
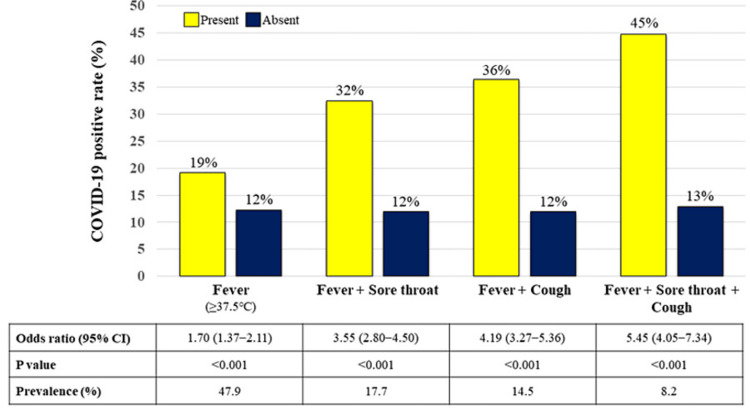
COVID-19 positive rate for simple three symptoms (fever, sore throat, and cough) Fever alone had a COVID-19 positive rate of 19%; however, when a sore throat, a cough, or both are added, the COVID-19 positive rate increased markedly. Conversely, COVID-19 positive rates in the absence of symptoms were consistently low, 12%–13%.

High-risk exposure to patients with COVID-19 was an independent strong predictor for COVID-19 with a high odds ratio of 23.1 (Table 3). Multivariate analysis after adjusting for age and gender showed fever, sore throat, cough, and arthralgia as positive predictors for COVID-19. By contrast, runny nose/nasal congestion, diarrhea, nausea/vomiting, and shortness of breath were negative predictors of COVID-19 (Table 4). COVID-19 was more frequently identified in patients having fever (≥37.5℃) with a sore throat, a cough, or both. The COVID-19-positive rate reached approximately half (45%) when three symptoms were present (Figure 3).

## Discussion

Using real-world data from the fever outpatient setting, this study revealed the following: (1) high-risk exposure to COVID-19 patients was at very high risk of developing COVID-19; (2) besides fever, sore throat, and cough were very common symptoms in COVID-19; (3) the combination of these three symptoms could easily predict COVID-19.

Symptoms in patients with COVID-19

Smith et al. previously reported that loss of taste or smell, fever, and cough are useful symptoms for the diagnosis of COVID-19 [9]. Similarly, Bhattacharya et al. reported that fever and loss of smell are important predictors for the diagnosis of COVID-19 [10]. However, it should be noted that different clinical characteristics were reported for each epidemic strain of COVID-19 [11].

In the present study, a loss of taste and smell, which were reported as important features in the early stages of the COVID-19 epidemic, were present in only 1.7% and 1.0% of patients, respectively, and were comparable with those without COVID-19. Most of the patients with COVID-19 in this study were detected during the Omicron wave, which may be affecting their symptoms. In fact, Laracy et al. reported that the symptoms of COVID-19 in the Omicron wave had a higher frequency of asymptomatic and sore throat, but a lower frequency of loss of taste and smell compared to the wave prior to the Omicron [11]. Therefore, we need to reanalyze and update the symptom-based prediction of COVID-19 as soon as possible within the wave period when a new SARS-CoV-2 mutant strain becomes epidemic.

High-risk exposure to patients with COVID-19

Close contact with patients with COVID-19 is a strong predictor of COVID-19 [5,6,12]. Ishii et al. reported using data from an outpatient clinic in Japan that 44.5% of COVID-19-positive patients had a history of close contact, significantly higher than the 26.0% of COVID-19-negative patients [13]. In this study, high-risk exposure to patients with COVID-19 had a very high odds ratio, consistent with previous reports. These may be attributed to the high infectivity of SARS-CoV-2, especially in the Omicron wave.

The CoVIDA study is the largest epidemiological study conducted in Colombia to investigate risk factors for SARS-CoV-2 transmission in close contact with adults. The study revealed that close contact with spouses and relatives increased the risk of COVID-19 infection with a 3.9- and 1.9-odds ratio, respectively, compared with non-relatives [2]. Madewell et al. previously reported that (1) the household secondary infection rate in COVID-19 was 16.6%, which was higher than that of SARS-CoV-1, which was 7.5% and (2) secondary household transmission of COVID-19 from a spouse was about twice as common as transmissions from other family contacts [14]. Luu et al. previously investigated the risk factors for SARS-CoV-2 transmission using a survey conducted across 66 countries and frequent close contact with colleagues, the habit of hugging when greeting is reported to be a risk for SARS-CoV-2 transmission [15]. Furthermore, studies have reported that close contact with symptomatic COVID-19 patients has a higher rate of secondary infection than with asymptomatic COVID-19 patients [2,14].

Thus, individuals with a history of close contact especially with symptomatic COVID-19 patients in the household should be carefully monitored because SARS-CoV-2 infectivity is further increased with the Omicron variant. Furthermore, community-acquired COVID-19 infections with unknown contact histories are increasing. Early suspicion of COVID-19 infection by simple symptoms might lead to proactive COVID-19 testing and help in early diagnosis and social isolation.

Limitations

This study has several limitations. First, a retrospective, single-center analysis was conducted. Therefore, the results need to be prospectively confirmed at other hospitals and communities. Second, symptoms may differ among SARS-CoV-2 strains. During the study period, 89% (364/410) of COVID-19-positive patients occurred in the Omicron phase. The small number of COVID-19-positive patients of other subtypes makes statistical analysis difficult. Moreover, COVID-19 subtypes were inferred from the epidemic period, and subtypes were not confirmed by testing. Therefore, further verification is needed to address the issue. Third, vaccination history for COVID-19 may have affected the results. However, vaccine efficacy is affected by the number of doses, the length of time since the last dose, and the type of vaccine. To address the issue, detailed studies focusing on vaccine efficacy are needed. Fourth, this study did not systematically measure oxygen levels or symptom severity because the data were obtained in a fever outpatient setting and did not include severe cases that would have been transported to the emergency room. Therefore, it is difficult to determine the exact severity of the illness.

## Conclusions

In the present study, we found using an outpatient clinic data in Japan that the presence of fever (≥37.5℃) with a sore throat, a cough, or both could be important predictors for the diagnosis of COVID-19. Our results suggested that the simple symptom-based COVID-19 prediction might be useful, which is accessible to the general public due to its simplicity. Moreover, close contact to COVID-19 infected patients is a strong predictor for the diagnosis of COVID-19. Thus, proactive COVID-19 testing of symptomatic individuals especially with a history of close contact to COVID-19 infected patients may contribute to early diagnosis and isolation, thereby preventing the spread of infection. Our results may reflect the characteristics of the Omicron strain, the current predominant strain of COVID-19 infection. However, symptoms and infectivity may vary from strain to strain, and thus newer COVID-19 variants may need to be reexamined.
